# Robust Distributed Kalman Filtering: On the Choice of the Local Tolerance

**DOI:** 10.3390/s20113244

**Published:** 2020-06-07

**Authors:** Alessandro Emanuele, Francesco Gasparotto, Giacomo Guerra, Mattia Zorzi

**Affiliations:** Department of Information Engineering, University of Padova, Via Gradenigo 6/B, 35131 Padova, Italy; alessandro.emanuele@studenti.unipd.it (A.E.); francesco.gasparotto@studenti.unipd.it (F.G.); giacomo.guerra.2@studenti.unipd.it (G.G.)

**Keywords:** distributed robust Kalman filtering, least favorable analysis, sensor networks

## Abstract

We propose a distributed Kalman filter for a sensor network under model uncertainty. The distributed scheme is characterized by two communication stages in each time step: in the first stage, the local units exchange their observations and then they can compute their local estimate; in the final stage, the local units exchange their local estimate and compute the final estimate using a diffusion scheme. Each local estimate is computed in order to be optimal according to the least favorable model belonging to a prescribed local ambiguity set. The latter is a ball, in the Kullback–Liebler topology, about the corresponding nominal local model. We propose a strategy to compute the radius, called local tolerance, for each local ambiguity set in the sensor network, rather than keep it constant across the network. Finally, some numerical examples show the effectiveness of the proposed scheme.

## 1. Introduction

Modern problems involve a large number of sensors forming a sensor network and taking measurements from which we would like to infer quantities not accessible to observation possibly at each node location. These problems can be classified as filtering problems whose solution is given by the Kalman filter. On the other hand, its implementation is very expensive in terms of data transmission; indeed, we require that all sensors can exchange their measurements. Such a limitation disappears by considering distributed filtering [1,2,3,4,5,6,7,8,9]. The key idea is that the communication among the nodes is limited.

In the simplest distributed strategy, the state estimate of a node (i.e., local unit) is computed by using only the observations from its neighbors. Such a strategy, however, appears to be not very effective. A remarkable progress has been reached by distributed Kalman filtering with consensus [10,11,12,13] and diffusion strategies [14,15]. These distributed approaches are characterized by several communication stages during each time step. For instance, in the first stage, the local units exchange their observations and then they can compute their local estimate; in the final stage, the local units exchange their local estimate and compute the final estimate using a consensus or a diffusion scheme. Many important challenges have been addressed in distributed filtering. For instance, the issues about limited observability, network topologies that restrict allowable communications, and communication noises between sensors are considered in [16]; the case in which the sensor network is subject to transmission delays is considered in [17]; and the cases of missing measurements and absence of communication among the nodes are analyzed in [18,19], respectively. It is worth observing that distributed state estimation can be performed also through different principles [20,21,22,23,24,25]. For instance, each node can transmit its measurements to a fusion center and then the latter computes the state estimate.

An important aspect in filtering applications is that the nominal model does not correspond to the actual one. Risk sensitive Kalman filtering [26,27,28] addresses this problem by penalizing large errors. The severity of this penalization is tuned by the so-called risk sensitivity parameter: the larger the risk sensitivity parameter is, the more large errors are penalized. A refinement of these filters is given by robust Kalman filtering where the uncertainty is expressed incrementally [29,30,31,32]. More precisely, for each time step, the state estimator minimizes the prediction error according to the least favorable model belonging to a prescribed ambiguity set. The latter is a ball in the Kullback–Leibler (KL) topology whose center is the nominal model. The radius of this ball is called tolerance and it represents the discrepancy budget between the actual and the nominal model allowed for the corresponding time step. It is worth noting that the ambiguity set can be formed also by using different types of divergence (see, for instance, [33,34,35]).

The problem of distributed Kalman filtering under model uncertainty has been considered as well (see, e.g., [36,37,38]). In the present paper, we consider the distributed robust Kalman filter with diffusion step proposed in [39]. Here, the local estimate of each node is computed by using the robust Kalman filter in [29]. In this scenario, the least favorable model is the one used to compute the robust Kalman filter of the global model. Accordingly, we have one ambiguity set corresponding to the global model, which contains the actual model, and the local ambiguity sets corresponding to the nodes of the network. The centers of those balls are known because they are given by the nominal model. The local tolerances corresponding to the local ambiguity sets of the nodes are set equal to the one of the global ambiguity set. On the other hand, the local ambiguity set of a node corresponds to a local model which is just a part of the global model. Accordingly, taking all the local tolerances uniform across the network and equal to the global one may not be the best choice.

The main contribution of this paper is to propose a robust distributed Kalman filter as in [39] where the local tolerance for each node is customized. In this way, the local tolerance is non-uniform and time-varying across the network. We show through some simulation studies that the performance of the predictions is improved. Moreover, we show that, if the tolerance corresponding to the global ambiguity set is sufficiently small, then the local tolerances across the network are constant in the steady-state condition. Accordingly, it is also possible to simplify the distributed scheme by replacing the time-varying tolerances with the steady-state values.

The organization of this paper is as follows. In Section 2, we provide the background about robust Kalman filtering whose uncertainty is expressed incrementally. In Section 3, the distributed robust Kalman filter with local uniform tolerance is reviewed. In Section 4, we introduce the distributed robust Kalman filter with non-uniform local tolerance. In Section 5, we perform some numerical experiments to check the performance of the proposed distributed scheme. In Section 6, we propose an efficient approximation of the distributed robust Kalman filter with non-uniform local tolerance. Finally, in Section 7, we draw the conclusions.

*Notation*: ${\left[a_{ij}\right]}_{ij}$ denotes the matrix having entry $a_{ij}$ in position (i,j); $A^{T}$ is the transposition of matrix *A*; and A>0(A≥0) means that matrix *A* is positive (semi)-definite. $\mathrm{diag}(A_{1}\cdots A_{n})$ denotes a block-diagonal matrix whose blocks in the main block diagonal are $A_{1}\cdots A_{n}$. Given a squared matrix *A*, tr(A) and |A| denote the trace and the determinant of *A*, respectively. Given two matrices *A* and *B*, A⊗B denotes their Kronecker product. x∼N(m,K) means that *x* is a Gaussian random vector with mean *m* and covariance matrix *K*.

## 2. Background

In this section, we review the robust Kalman filter proposed in [29], which represents the “building block” used throughout the paper. Consider the nominal state-space model

(1) $$\begin{array}{cc} x_{t+1} & =Ax_{t}+\Gamma_{B}u_{t}+r_{t}\\ y_{t} & =Cx_{t}+\Gamma_{D}u_{t} \end{array}$$

where $A\in R^{n\times n}$, $\Gamma_{B}\in R^{n\times n+pN}$, $C\in R^{pN\times n}$, $\Gamma_{D}\in R^{pN\times n+pN}$, $x_{t}$ is the state process, $y_{t}$ is the observation process, $u_{t}$ is normalized white Gaussian noise (WGN), and $r_{t}$ is a deterministic signal. It is assumed that $u_{t}$ is independent from the initial state $x_{0}∼N\left({\hat{x}}_{0},V_{0}\right)$. We also assume that the noise entering in the state process and the one entering in the observation process are independent, i.e., we assume that $\Gamma_{B}\Gamma_{D}^{T}=0$. Finally, the state-space model in Equation (1) is considered to be reachable and observable. Let $\phi_{t}\left(z_{t}|x_{t}\right)$ denote the nominal transition probability density of $z_{t}:={\left[x_{t+1}^{T}y_{t}^{T}\right]}^{T}$ given $x_{t}$. Notice that $\phi_{t}\left(z_{t}|x_{t}\right)$ is Gaussian by construction and it is straightforwardly given by Equation (1).

We assume that the (unknown) actual transition probability ${\tilde{\phi }}_{t}\left(z_{t}|x_{t}\right)$ belongs to the ambiguity set which is a closed ball centered in $\phi_{t}\left(z_{t}|x_{t}\right)$ in the KL topology:

(2) $$B_{t}:=\left\{{\tilde{\phi }}_{t}\;s.t.\;\tilde{E}\left.\left[\log\left(\frac{{\tilde{\phi }}_{t}}{\phi_{t}}\right)\right|Y_{t-1}\right]\leq c\right\}$$

with

$$\tilde{E}\left.\left[\log\left(\frac{{\tilde{\phi }}_{t}}{\phi_{t}}\right)\right|Y_{t-1}\right]:=\;\int \int \;{\tilde{\phi }}_{t}\left(z_{t}|x_{t}\right){\overset{ˇ}{f}}_{t}\left(x_{t}|Y_{t-1}\right)\log\left(\frac{{\tilde{\phi }}_{t}\left(z_{t}|x_{t}\right)}{\phi_{t}\left(z_{t}|x_{t}\right)}\right)dz_{t}dx_{t},$$

$Y_{t-1}:=\left\{y_{s},\;s=0\cdots t-1\right\}$, and ${\overset{ˇ}{f}}_{t}\left(x_{t}|Y_{t-1}\right)∼N\left({\hat{x}}_{t},V_{t}\right)$ is defined as the actual conditional probability density of $x_{t}$ given $Y_{t-1}$. The mismatch modeling budget allowed for each time step is represented by the parameter c>0, which is called tolerance. The robust estimator of $x_{t+1}$ given $Y_{t}$ for the nominal model in Equation (1) is given by solving the following minimax problem:

(3) $${\hat{x}}_{t+1}=\underset{g_{t}\in G_{t}}{\mathrm{argmin}}\max_{{\tilde{\phi }}_{t}\in B_{t}}\tilde{E}\left.\left[{∥x_{t+1}-g_{t}\left(y_{t}\right)∥}^{2}\right|Y_{t-1}\right]$$

where $G_{t}$ is the set of all estimators $g_{t}$ whose variance is finite under any model in the ambiguity set $B_{t}$,

$$\begin{array}{c} \tilde{E}\left.\left[{∥x_{t+1}-g_{t}\left(y_{t}\right)∥}^{2}\right|Y_{t-1}\right]:=\;\int \int \;{∥x_{t+1}-g_{t}\left(y_{t}\right)∥}^{2}{\tilde{\phi }}_{t}\left(z_{t}|x_{t}\right){\overset{ˇ}{f}}_{t}\left(x_{t}|Y_{t-1}\right)dz_{t}dx_{t} \end{array}$$

is the estimation error under the transition density ${\tilde{\phi }}_{t}\left(z_{t}|x_{t}\right)$. In [29], it is proved that the estimator solution to the problem in Equation (3) has the following Kalman-like structure:

(4) $$\begin{array}{cc} G_{t} & =AV_{t}C^{T}{\left(CV_{t}C^{T}+\Gamma_{D}\Gamma_{D}^{T}\right)}^{-1}\\ {\hat{x}}_{t+1} & =A{\hat{x}}_{t}+G_{t}\left(y_{t}-C{\hat{x}}_{t}\right)+r_{t}\\ P_{t+1} & =A{\left(V_{t}^{-1}+C^{T}{\left(\Gamma_{D}\Gamma_{D}^{T}\right)}^{-1}C\right)}^{-1}A^{T}+\Gamma_{B}\Gamma_{B}^{T}\\ \mathrm{Find}\; & \theta_{t}\;s.t.\;\gamma \left(P_{t+1},\theta_{t}\right)=c\\ V_{t+1} & ={\left(P_{t+1}^{-1}-\theta_{t}I\right)}^{-1} \end{array}$$

where

$$\gamma (P,\theta ):=\log\det\left(I-\theta P\right)+\mathrm{tr}({\left(I-\theta P\right)}^{-1}-I).$$

Parameter $\theta_{t}>0$ is called risk sensitivity parameter. It is worth noting that, given P>0 and c>0, the equation γ(P,θ)=c always admits a unique solution in θ and such that: θ>0, $P^{-1}-\theta I>0$. In the special case that c=0, i.e., the nominal model coincides with the actual model, then $\theta_{t}=0$ and thus Equation (4) degenerates in the usual Kalman filter.

**Remark** **1.** *It is worth noting that the robust Kalman filter is well defined also in the case that the ambiguity set Equation (2) is defined by a time-varying tolerance, i.e., $c_{t}$ instead of c. However, we prefer to keep c constant in Equation (3) because in the following we assume that the actual (global) model is the solution to Equation (3) with constant tolerance c, in order to simplify the setup.*

## 3. Distributed Robust Kalman Filtering with Uniform Local Tolerance

In this section, we review the distributed robust Kalman filter presented in [39]. Consider a network made by *N* sensors. The latter are connected if they can communicate with each other. Accordingly, every sensor *k* has a set of neighbors which is denoted by $N_{k}$. In particular, $k\in N_{k}$ that is each node is connected with itself. The number of neighbors of node *k* is denoted by $n_{k}$. The corresponding N×N adjacency matrix $J={\left[j_{lk}\right]}_{lk}$ is defined as

$$j_{lk}:=\left\{\begin{array}{cc} 1, & \;\mathrm{if}\;l\in N_{k}\\ 0, & \;\mathrm{otherwise}. \end{array}\right.$$

We assume that every node collects a measurement $y_{k,t}\in R^{p}$ at time *t* and the corresponding nominal state-space model is

(5) $$\begin{array}{cc} x_{t+1} & =Ax_{t}+Bw_{t}+r_{t}\\ y_{k,t} & =C_{k}x_{t}+D_{k}v_{k,t}\;k=1\ldots N \end{array}$$

where $w_{t}$ and $v_{k,t}$, with k=1…N, are independent normalized WGNs. It is worth noting that the actual state-space model for each node is unknown. By stacking Equation (5) for every *k*, it is possible to rewrite such sensor network as Equation (1) where:

(6) $$\begin{array}{c} y_{t}=\left[\begin{array}{c} y_{1,t}\\ \vdots \\ y_{N,t} \end{array}\right],\;u_{t}=\left[\begin{array}{c} w_{t}\\ v_{t} \end{array}\right],\;v_{t}=\left[\begin{array}{c} v_{1,t}\\ \vdots \\ v_{N,t} \end{array}\right]\\ \Gamma_{B}=\left[\begin{array}{cc} B & 0 \end{array}\right],\;\Gamma_{D}=\left[\begin{array}{cc} 0 & D \end{array}\right]\\ C=\left[\begin{array}{c} C_{1}\\ \vdots \\ C_{N} \end{array}\right],\;D=\mathrm{diag}\left(D_{1},\ldots ,D_{N}\right). \end{array}$$

Accordingly, Equation (4) represents the centralized robust Kalman filter. Defining $R:=DD^{T}$, $R_{l}:=D_{l}D_{l}^{T}$ with l=1…N, and

$$S_{tot}:=C^{T}R^{-1}C=\sum_{l=1}^{N}C_{l}^{T}R_{l}^{-1}C_{l},$$

the Kalman gain for Equation (5) becomes, using the matrix inversion lemma,

(7) $$G_{t}=A{\left(V_{t}^{-1}+S_{tot}\right)}^{-1}C^{T}R^{-1}.$$

Since the nominal model in Equation (5) does not coincide with the actual one and each node *k* can only exploit information shared by its neighbors $l\in N_{k}$, the aim of distributed robust Kalman filtering is to compute a prediction ${\hat{x}}_{k,t}$ of the state $x_{t}$ for every node *k* by using only the local information, taking into account the model uncertainty. In the case that the node *k* has access to all measurements across all the nodes in the network, then ${\hat{x}}_{k,t}$ coincides with Equation (4) which can be written, using the parameterization in Equations (6) and (7) as

(8) $$\begin{array}{cc} {\hat{x}}_{k,t+1} & =A{\hat{x}}_{k,t}+A{\left(V_{k,t}^{-1}+S_{tot}\right)}^{-1}\sum_{l=1}^{N}C_{l}^{T}R_{l}^{-1}\left(y_{l,t}-C_{l}{\hat{x}}_{k,t}\right)+r_{t}\\ P_{k,t+1} & =A{\left(V_{k,t}^{-1}+S_{tot}\right)}^{-1}A^{T}+BB^{T}\\ \mathrm{Find}\; & \theta_{k,t}\;s.t.\;\gamma \left(P_{k,t+1},\theta_{k,t}\right)=c\\ V_{k,t+1} & ={\left(P_{k,t+1}^{-1}-\theta_{k,t}I\right)}^{-1} \end{array}$$

where ${\hat{x}}_{k,t}={\hat{x}}_{t}$, $P_{k,t}=P_{t}$, $V_{k,t}=V_{t}$, and $\theta_{k,t}=\theta_{t}$. In the case that not all the measurements in the network are accessible to node *k*, then the target is to compute a state prediction ${\hat{x}}_{k,t}$ of $x_{t}$ which is as similar as possible to the global state prediction.

Assume that the node *k* can collect the measurements from its neighbors $N_{k}$. Then, the corresponding local nominal state-space model is

(9) $$\begin{array}{cc} x_{t+1} & =Ax_{t}+Bw_{t}+r_{t}\\ y_{l,t} & =C_{l}x_{t}+D_{l}v_{l,t},\;l\in N_{k}. \end{array}$$

The latter can be rewritten in the compact form

(10) $$\begin{array}{cc} x_{t+1} & =Ax_{t}+\Gamma_{B}u_{k,t}^{\mathrm{loc}}+r_{t}\\ y_{k,t}^{\mathrm{loc}} & =C_{k}^{\mathrm{loc}}x_{t}+\Gamma_{D_{k}^{\mathrm{loc}}}u_{k,t}^{\mathrm{loc}} \end{array}$$

where $u_{k,t}^{\mathrm{loc}}={\left[w_{t}^{T}\;{\left(v_{k,t}^{\mathrm{loc}}\right)}^{T}\right]}^{T}$ is the input noise and $y_{k,t}^{\mathrm{loc}}$ is the output; $v_{k,t}^{\mathrm{loc}}$ and $y_{k,t}^{\mathrm{loc}}$ are given by stacking $v_{l,t}$ and $y_{l,t}$, with $l\in N_{k}$, respectively. Moreover, $C_{k}^{\mathrm{loc}}$ is given by stacking $C_{l}$ with $l\in N_{k}$, $\Gamma_{D^{\mathrm{loc}}}=\left[0\;D_{k}^{\mathrm{loc}}\right]$ and $D_{k}^{\mathrm{loc}}$ is a block diagonal matrix whose main blocks are $D_{l}$ with $l\in N_{k}$. In addition, defining $R_{k}^{\mathrm{loc}}:=D_{k}^{\mathrm{loc}}{\left(D_{k}^{\mathrm{loc}}\right)}^{T}$ and $S_{k}:={\left(C_{k}^{\mathrm{loc}}\right)}^{T}{\left(R_{k}^{\mathrm{loc}}\right)}^{-1}C_{k}^{\mathrm{loc}}$ it results that

$$S_{k}=\sum_{l\in N_{k}}C_{l}^{T}R_{l}^{-1}C_{l}.$$

We conclude that the one-step ahead predictor of $x_{t}$ at node *k* is similar to the one in Equation (8) but now we need to discard the terms for which $l\notin N_{k}$. It is worth noting that the latter represents an intermediate local prediction of $x_{t+1}$ at node *k*, and it is denoted as $\psi_{k,t+1}$. Allowing that the connected nodes can exchange their intermediate estimates, then each node can update the prediction at node *k* in terms of both $\psi_{k,t+1}$ and $\psi_{l,t+1}$ with $l\in N_{k}$. More precisely, consider a matrix $W={\left[w_{lk}\right]}_{lk}\in R^{N\times N}$ such that

(11) $$\begin{array}{c} w_{lk}\geq 0\;\mathrm{and}\;w_{lk}=0\;\mathrm{if}\;l\notin N_{k}\\ \sum_{l\in N_{k}}w_{lk}=1. \end{array}$$

Therefore, the final predicted state at node *k* is given by means of the so-called diffusion step [14]:

$${\hat{x}}_{k,t+1}=\sum_{l\in N_{k}}w_{lk}\psi_{l,t+1}.$$

To sum up, in the diffusion scheme, each local unit uses the measurements and the intermediate local predictions from its neighbors. The resulting scheme is explained through Algorithm 1.
**Algorithm 1 **Distributed robust Kalman filter with uniform local tolerance at time *t*.**Input:**${\hat{x}}_{k,t},V_{k,t},y_{k,t},W={\left[w_{lk}\right]}_{lk}$ with k=1…N**Output:**${\hat{x}}_{k,t+1},V_{k,t+1}$ with k=1…N**Incremental step.** Compute at every node *k*:
(12)$$\begin{array}{cc} \psi_{k,t+1} & =A{\hat{x}}_{k,t}+A{\left(V_{k,t}^{-1}+S_{k}\right)}^{-1}\sum_{l\in N_{k}}C_{l}^{T}R_{l}^{-1}\left(y_{l,t}-C_{l}{\hat{x}}_{k,t}\right)+r_{t}\\ P_{k,t+1} & =A{\left(V_{k,t}^{-1}+S_{k}\right)}^{-1}A^{T}+BB^{T}\\ \mathrm{Find}\; & \theta_{k,t}\;s.t.\;\gamma \left(P_{k,t+1},\theta_{k,t}\right)=c\\ V_{k,t+1} & ={\left(P_{k,t+1}^{-1}-\theta_{k,t}I\right)}^{-1} \end{array}$$**Diffusion step.** Compute at every node *k*:
(13)$${\hat{x}}_{k,t+1}=\sum_{l\in N_{k}}w_{lk}\psi_{l,t+1}$$

It is worth noting that $\psi_{k,t}$ is computed by using the robust Kalman scheme in Equation (4) applied to the local model in Equation (10). In addition, *c* is the same for any node that is *c* takes a uniform value over the sensor network. In particular, the tolerance *c* is the same for both the centralized and the distributed Kalman filter. This strategy for the selection of the tolerance does not ensure that the least favorable model computed at node *k* is compatible with the one of the centralized filter. However, in the case of large deviations of the least favorable model corresponding to the centralized problem, it is very likely that the predictor at node *k* using Algorithm 1 is better than the one which assumes that the nominal and actual models coincide. Finally, in the case that c=0, i.e., the nominal model coincides with the actual one, Algorithm 1 boils down to the distributed Kalman filter with diffusion step in [14].

## 4. Distributed Robust Kalman Filtering with Non-Uniform Local Tolerance

We investigate the possibility to assign a possibly different local tolerance to each node that is the local tolerance is not uniform across the sensor network. Recall that the least favorable model is given by the minimax problem in Equation (3), with constant tolerance *c*, and the corresponding optimal estimator is given by the centralized robust Kalman filter in Equation (4).

Consider the centralized problem in Equation (3). Let

(14) $$\begin{array}{c} {\bar{f}}_{t}\left(z_{t}|Y_{t-1}\right)=\int \phi_{t}\left(z_{t}|x_{t}\right){\overset{ˇ}{f}}_{t}\left(x_{t}|Y_{t-1}\right)dx_{t} \end{array}$$

(15) $$\begin{array}{c} {\tilde{f}}_{t}\left(z_{t}|Y_{t-1}\right)=\int {\tilde{\phi }}_{t}\left(z_{t}|x_{t}\right){\overset{ˇ}{f}}_{t}\left(x_{t}|Y_{t-1}\right)dx_{t} \end{array}$$

denote the pseudo-nominal and the least favorable conditional probability densities of $z_{t}$ given the past observations $Y_{t-1}$, respectively. Recall that $\phi_{t}\left(z_{t}|x_{t}\right)$ is the nominal transition density of the state space model in Equation (1) and thus

(16) $$\begin{array}{c} \phi_{t}\left(z_{t}|x_{t}\right)∼N\left(\left[\begin{array}{c} Ax_{t}+r_{t}\\ Cx_{t} \end{array}\right],\left[\begin{array}{c} \Gamma_{B}\\ \Gamma_{D} \end{array}\right]\left[\begin{array}{cc} \Gamma_{B}^{T} & \Gamma_{D}^{T} \end{array}\right]\right). \end{array}$$

Since ${\overset{ˇ}{f}}_{t}\left(x_{t}|Y_{t-1}\right)∼N\left({\hat{x}}_{t},V_{t}\right)$, and in view of Equations (14) and (16), we have

$${\bar{f}}_{t}\left(z_{t}|Y_{t-1}\right)∼N\left(m_{z},K_{z_{t}}\right)$$

where

(17) $$m_{z_{t}}=\left[\begin{array}{c} A{\hat{x}}_{t}+r_{t}\\ C{\hat{x}}_{t} \end{array}\right],\;K_{z_{t}}=\left[\begin{array}{c} A\\ C \end{array}\right]V_{t}\left[\begin{array}{cc} A^{T} & C^{T} \end{array}\right]+\left[\begin{array}{c} \Gamma_{B}\\ \Gamma_{D} \end{array}\right]\left[\begin{array}{cc} \Gamma_{B}^{T} & \Gamma_{D}^{T} \end{array}\right].$$

In [29], it has been shown that the optimal solution ${\tilde{\phi }}_{t}^{0}\left(z_{t}|x_{t}\right)$ to Equation (3) is Gaussian. Accordingly, in view of Equation (15), the corresponding least favorable density of $z_{t}$ given $Y_{t-1}$ is Gaussian:

$${\tilde{f}}_{t}\left(z_{t}|Y_{t-1}\right)∼N\left({\tilde{m}}_{z_{t}},{\tilde{K}}_{z_{t}}\right).$$

It is clear then that the minimax problem in Equation (3) can be written by replacing $\phi_{t}\left(z_{t}|x_{t}\right)$ and ${\tilde{\phi }}_{t}\left(z_{t}|x_{t}\right)$ with ${\bar{f}}_{t}\left(z_{t}|Y_{t-1}\right)$ and ${\tilde{f}}_{t}\left(z_{t}|Y_{t-1}\right)$, respectively. Then, the equivalent minimax problem is

(18) $${\hat{x}}_{t+1}=\underset{g_{t}\in G_{t}}{\mathrm{argmin}}\max_{{\tilde{f}}_{t}\in {\bar{B}}_{t}}\int {∥x_{t+1}-g_{t}∥}^{2}{\tilde{f}}_{t}\left(z_{t}|Y_{t-1}\right)dz_{t}$$

where the ambiguity set is a ball about the pseudo-nominal density ${\bar{f}}_{t}\left(z_{t}|Y_{t-1}\right)$

$$\begin{array}{cc} {\bar{B}}_{t} & =\left\{{\tilde{f}}_{t}\left(z_{t}|Y_{t-1}\right)∼N\left({\tilde{m}}_{z_{t}},{\tilde{K}}_{z_{t}}\right)\;s.t.\;D_{KL}\left({\tilde{f}}_{t}∥{\bar{f}}_{t}\right)\leq c\right\} \end{array}$$

formed by the KL divergence between ${\tilde{f}}_{t}\left(z_{t}|Y_{t-1}\right)$ and ${\bar{f}}_{t}\left(z_{t}|Y_{t-1}\right)$:

$$\begin{array}{cc} D_{KL}\left({\tilde{f}}_{t}∥{\bar{f}}_{t}\right) & =\int {\tilde{f}}_{t}\left(z_{t}|Y_{t-1}\right)\log\left(\frac{{\tilde{f}}_{t}\left(z_{t}|Y_{t-1}\right)}{{\bar{f}}_{t}\left(z_{t}|Y_{t-1}\right)}\right)dz_{t}. \end{array}$$

Since ${\tilde{f}}_{t}\left(z_{t}|Y_{t-1}\right)$ and ${\bar{f}}_{t}\left(z_{t}|Y_{t-1}\right)$ are Gaussian distributed, we have

$$D_{KL}\left({\tilde{f}}_{t}∥{\bar{f}}_{t}\right)=\frac{1}{2}\left[∥m_{z_{t}}-{\tilde{m}}_{z_{t}}{∥}_{K_{z_{t}}^{-1}}^{2}-\log\left|{\tilde{K}}_{z_{t}}\right|+\log\left|K_{z_{t}}\right|+\mathrm{tr}\left({\tilde{K}}_{z_{t}}K_{z_{t}}^{-1}\right)-\left(n+p\right)\right].$$

It is well known that $D_{KL}\left({\tilde{f}}_{t}∥{\bar{f}}_{t}\right)$ also represents the negative log-likelihood of the model ${\bar{f}}_{t}$ under the actual model ${\tilde{f}}_{t}$, [40,41,42]. Accordingly, *c* represents an upper bound of the negative log-likelihood and it can be found as follows. Fix the nominal state space model (A,B,C,D) and collect the data $\left(y^{N},u^{N},x^{N}\right)$ where $y^{N}=\left\{y_{1}\ldots y_{N}\right\}$, $u^{N}=\left\{u_{1}\ldots u_{N}\right\}$, $x^{N}=\left\{x_{1}\ldots x_{N}\right\}$. Let $\ell (A,B,C,D;y^{N},u^{N},x^{N})$ be the negative log-likelihood of this nominal model using the collected data. Then, fix $c=\ell (A,B,C,D;y^{N},u^{N},x^{N})$. Clearly, we need to assume that the state is accessible to observation (or its estimate is reasonably good) to compute *c*.

**Theorem** **1** (Levy & Nikoukhah [30])**.** *Let ${\bar{f}}_{t}\left(z_{t}|Y_{t-1}\right)$ be the nominal density with mean $m_{z_{t}}$ and covariance matrix $K_{z_{t}}$ partitioned as*

$$K_{z_{t}}=\left[\begin{array}{cc} K_{x_{t+1}} & K_{x_{t+1},y_{t}}\\ K_{y_{t},x_{t+1}} & K_{y_{t}} \end{array}\right]$$

*according to the dimension of $x_{t+1}$ and $y_{t}$, respectively. The least favorable density ${\tilde{f}}_{t}^{0}\left(z_{t}|Y_{t-1}\right)$ solution to Equation (18) has mean and covariance matrix as follows:*

$${\tilde{m}}_{z_{t}}=m_{z_{t}},\;{\tilde{K}}_{z_{t}}=\left[\begin{array}{cc} {\tilde{K}}_{x_{t+1}} & K_{x_{t+1},y_{t}}\\ K_{y_{t},x_{t+1}} & K_{y_{t}} \end{array}\right].$$

*Let*

(19) $$\begin{array}{cc} P_{t+1} & =K_{x_{t+1}}-K_{x_{t+1},y_{t}}K_{y_{t}}^{-1}K_{y_{t},x_{t+1}}\\ V_{t+1} & ={\tilde{K}}_{x_{t+1}}-K_{x_{t+1},y_{t}}K_{y_{t}}^{-1}K_{y_{t},x_{t+1}} \end{array}$$

*denote the nominal and least favorable error covariance matrices of $x_{t+1}$ given $Y_{t}$. Then,*

(20) $$\begin{array}{c} V_{t+1}={\left(P_{t+1}^{-1}-\theta_{t}I\right)}^{-1} \end{array}$$

*and $\theta_{t}>0$ is the unique value for which*

(21) $$\begin{array}{c} D_{KL}\left({\tilde{f}}_{t}^{0}∥{\bar{f}}_{t}\right)=\frac{1}{2}\left[-\log\left|{\tilde{K}}_{z_{t}}\right|+\log\left|K_{z_{t}}\right|+\mathrm{tr}\left({\tilde{K}}_{z_{t}}K_{z_{t}}^{-1}\right)-\left(n+p\right)\right]=c. \end{array}$$

The above result provides a way to compute ${\tilde{f}}_{t}^{0}\left(z_{t}|Y_{t-1}\right)$. Indeed, once the centralized robust Kalman filter in Equation (4) has been computed, the mean and the covariance matrix of ${\tilde{f}}_{t}^{0}\left(z_{t}|Y_{t-1}\right)$ are given, in view of Equation (17), by 

(22) $$\begin{array}{c} {\tilde{m}}_{z_{t}}=\left[\begin{array}{c} A{\hat{x}}_{t}+r_{t}\\ C{\hat{x}}_{t} \end{array}\right],\;{\tilde{K}}_{z_{t}}=\left[\begin{array}{cc} V_{t+1}+K_{x_{t+1},y_{t}}K_{y_{t}}^{-1}K_{y_{t},x_{t+1}} & K_{x_{t+1},y_{t}}\\ K_{y_{t},x_{t+1}} & K_{y_{t}} \end{array}\right] \end{array}$$

where

$$\begin{array}{cc} K_{x_{t+1},y_{t}} & =AV_{t}C^{T}\\ K_{y_{t}} & =CV_{t}C^{T}+\Gamma_{D}\Gamma_{D}^{T}. \end{array}$$

From ${\bar{f}}_{t}\left(z_{t}|Y_{t-1}\right)$ and ${\tilde{f}}_{t}^{0}\left(z_{t}|Y_{t-1}\right)$, we can compute the nominal and least favorable density for each node. Consider the state-space model in Equation (10) for node *k*. Let $z_{k,t}:={\left[\;x_{t+1}^{T}\;{\left(y_{k,t}^{\mathrm{loc}}\right)}^{T}\;\right]}^{T}$. Then, the nominal transition probability at node *k*, in view of Equation (10), is

(23) $$\begin{array}{c} \phi_{k,t}\left(z_{k,t}|x_{t}\right)∼N\left(\left[\begin{array}{c} Ax_{t}+r_{t}\\ C_{k}^{\mathrm{loc}}x_{t} \end{array}\right],\left[\begin{array}{c} \Gamma_{B}\\ \Gamma_{D_{k}^{\mathrm{loc}}} \end{array}\right]\left[\begin{array}{cc} \Gamma_{B}^{T} & \Gamma_{D_{k}^{\mathrm{loc}}}^{T} \end{array}\right]\right). \end{array}$$

Then,

$$\begin{array}{c} {\bar{f}}_{k,t}\left(z_{k,t}|Y_{t-1}\right)=\int \phi_{k,t}\left(z_{k,t}|x_{t}\right){\overset{ˇ}{f}}_{t}\left(x_{t}|Y_{t-1}\right)dx_{t} \end{array}$$

denotes the pseudo-nominal conditional probability density of $z_{k,t}$ given the past observations $Y_{t-1}$ at node *k*. Since ${\overset{ˇ}{f}}_{t}\left(x_{t}|Y_{t-1}\right)∼N\left({\hat{x}}_{t},V_{t}\right)$, and in view of Equation (23), we have

$$\begin{array}{cc} {\bar{f}}_{k,t}\left(z_{k,t}|Y_{t-1}\right) & ∼N\left(m_{z_{k,t}},K_{z_{k,t}}\right) \end{array}$$

where

(24) $$\begin{array}{cc} m_{z_{k,t}} & =\left[\begin{array}{c} A{\hat{x}}_{t}+r_{t}\\ C_{k}^{\mathrm{loc}}{\hat{x}}_{t} \end{array}\right]\\ K_{z_{k,t}} & =\left[\begin{array}{cc} K_{x_{t+1}} & K_{x_{t+1},y_{k,t}}\\ K_{y_{k,t},x_{t+1}} & K_{y_{k,t}} \end{array}\right] \end{array}$$

and

(25) $$\begin{array}{cc} K_{x_{t+1},y_{k,t}} & =AV_{t}{\left(C_{k}^{\mathrm{loc}}\right)}^{T}\\ K_{y_{k,t}} & =C_{k}^{\mathrm{loc}}V_{t}{\left(C_{k}^{\mathrm{loc}}\right)}^{T}+\Gamma_{D_{k}^{\mathrm{loc}}}\Gamma_{D_{k}^{\mathrm{loc}}}^{T}. \end{array}$$

Such a result is not surprising, indeed ${\bar{f}}_{k,t}\left(z_{k,t}|Y_{t-1}\right)$ is given by marginalizing ${\bar{f}}_{t}\left(z_{t}|Y_{t-1}\right)$ with respect to $y_{l,t}$ with $l\notin N_{k}$. Roughly speaking, this means that $m_{z_{k,t}}$, $K_{x_{t+1},y_{k,t}}$ and $K_{y_{k,t}}$ are obtained from $m_{z_{t}}$, $K_{x_{t+1},y_{t}}$ and $K_{y_{t}}$ as follows:

- $m_{z_{k,t}}$ is the vector obtained from $m_{z_{t}}$ by deleting the elements from pl−p+1 to pl for any $l\notin N_{k}$.
- $K_{x_{t+1},y_{k,t}}$ is the matrix obtained from $K_{x_{t+1},y_{t}}$ by deleting the columns from pl−p+1 to pl for any $l\notin N_{k}$.
- $K_{y_{k,t}}$ is the matrix obtained from $K_{y_{t}}$ by deleting the rows and the columns from pl−p+1 to pl for any $l\notin N_{k}$.

Accordingly, we can compute the least favorable density at node *k*, say ${\tilde{f}}_{k,t}^{0}\left(z_{k,t}|Y_{t-1}\right)$, by marginalizing ${\tilde{f}}_{t}^{0}\left(z_{t}|Y_{t-1}\right)$ with respect to $y_{l,t}$ with $l\notin N_{k}$. Therefore, we have

$$\begin{array}{cc} {\tilde{f}}_{k,t}^{0}\left(z_{k,t}|Y_{t-1}\right) & ∼N\left(m_{z_{k,t}},{\tilde{K}}_{z_{k,t}}\right) \end{array}$$

with

$${\tilde{K}}_{z_{k,t}}=\left[\begin{array}{cc} {\tilde{K}}_{x_{t+1}} & K_{x_{t+1},y_{k,t}}\\ K_{y_{k,t},x_{t+1}} & K_{y_{k,t}} \end{array}\right]=\left[\begin{array}{cc} V_{t+1}+K_{x_{t+1},y_{t}}K_{y_{t}}^{-1}K_{y_{t},x_{t+1}} & K_{x_{t+1},y_{k,t}}\\ K_{y_{k,t},x_{t+1}} & K_{y_{k,t}} \end{array}\right]$$

where in the last equality we exploit Equation (22). It remains to design the robust filter to compute the intermediate prediction $\psi_{k,t+1}$.

**Remark** **2.** *At this point, it is worth doing a digression about Algorithm 1. The intermediate prediction at node k is the solution to the following minimax problem*

(26) $$\psi_{k,t+1}=\underset{g_{k,t}\in G_{k,t}}{\mathrm{argmin}}\max_{{\tilde{f}}_{k,t}\in {\bar{B}}_{k,t}}\int {∥x_{t+1}-g_{k,t}\left(y_{k,t}^{\mathrm{loc}}\right)∥}^{2}{\tilde{f}}_{k,t}\left(z_{k,t}|Y_{t-1}\right)dz_{k,t}$$

*where*

(27) $$\begin{array}{cc} {\bar{B}}_{k,t}:= & \left\{{\tilde{f}}_{k,t}\;s.t.\;D_{KL}\left({\tilde{f}}_{k,t}∥{\bar{f}}_{k,t}\right)\leq c\right\} \end{array}$$

*and $G_{k,t}$ is the set of all estimators $g_{k,t}$ whose variance is finite under any model in the ambiguity set ${\bar{B}}_{k,t}$. Moreover, in view of Theorem 1, the least favorable density ${\tilde{f}}_{k,t}^{★}\left(z_{k,t}|Y_{t-1}\right)$ solution to Equation (26) is such that $D_{KL}\left({\tilde{f}}_{k,t}^{★}∥{\bar{f}}_{k,t}\right)=c$. It is worth noting that the best estimator at node k would be the one constructed from ${\tilde{f}}_{k,t}^{0}$. On the other hand, the problem in Equation (26) implies neither ${\tilde{f}}_{t,k}^{0}={\tilde{f}}_{t,k}^{★}$ nor $D_{KL}\left({\tilde{f}}_{k,t}^{★}∥{\bar{f}}_{k,t}\right)=D_{KL}\left({\tilde{f}}_{k,t}^{0}∥{\bar{f}}_{k,t}\right)$.*

Clearly, one would design the intermediate estimator at node *k* by using ${\tilde{f}}_{k,t}^{0}$. However, the latter is not available at node *k*, and it is only known by a “central unit”, i.e., a unit knowing the global model, but neither collecting measurements nor computing predictions. Moreover, the transmission of the mean and the covariance matrix of ${\tilde{f}}_{k,t}^{0}$ would be more expensive in terms of transmission costs. As alternative, we can consider a minimax problem whose least favorable model ${\tilde{f}}_{k,t}^{★}$ is such that $D_{KL}\left({\tilde{f}}_{k,t}^{★}∥{\bar{f}}_{k,t}\right)=D_{KL}\left({\tilde{f}}_{k,t}^{0}∥{\bar{f}}_{k,t}\right)$:

(28) $$\psi_{k,t+1}=\underset{g_{k,t}\in G_{k,t}}{\mathrm{argmin}}\max_{{\tilde{f}}_{k,t}\in {\bar{B}}_{k,t}}\int {∥x_{t+1}-g_{k,t}\left(y_{k,t}^{\mathrm{loc}}\right)∥}^{2}{\tilde{f}}_{k,t}\left(z_{k,t}|Y_{t-1}\right)dz_{k,t}$$

where

(29) $$\begin{array}{cc} {\bar{B}}_{k,t}:= & \left\{{\tilde{f}}_{k,t}\;s.t.\;D_{KL}\left({\tilde{f}}_{k,t}∥{\bar{f}}_{k,t}\right)\leq c_{k,t}\right\}, \end{array}$$

(30) $$\begin{array}{cc} c_{k,t}:= & \frac{1}{2}\left[-\log\left|{\tilde{K}}_{z_{k,t}}\right|+\log\left|K_{z_{k,t}}\right|+\mathrm{tr}\left({\tilde{K}}_{z_{k,t}}K_{z_{k,t}}^{-1}\right)-\left(n+p_{k}\right)\right], \end{array}$$

and $p_{k}$ coincides with the number of rows of $C_{k}^{\mathrm{loc}}$. Under the above scheme, the central unit only transmits the local tolerance to each node in the network. The procedure which implements this optimized strategy of distributed robust Kalman filtering is outlined in Algorithm 2.
**Algorithm 2  **Distributed robust Kalman filter with non-uniform local tolerance at time *t*.**Input:**${\hat{x}}_{k,t},V_{k,t},y_{k,t},W={\left[w_{lk}\right]}_{lk}$ with k=1…N**Output:**${\hat{x}}_{k,t+1},V_{k,t+1}$ with k=1…N**Tolerance update.** Using the nominal global model, the central unit computes for every node *k*:
(31)$c_{k,t}=\frac{1}{2}\left[-\log\left|{\tilde{K}}_{z_{k,t}}\right|+\log\left|K_{z_{k,t}}\right|+\mathrm{tr}\left({\tilde{K}}_{z_{k,t}}K_{z_{k,t}}^{-1}\right)-\left(n+p_{k}\right)\right]$**Incremental step.** Compute at every node *k*:
(32)$$\begin{array}{cc} \psi_{k,t+1} & =A{\hat{x}}_{k,t}+A{\left(V_{k,t}^{-1}+S_{k}\right)}^{-1}\sum_{l\in N_{k}}C_{l}^{T}R_{l}^{-1}\left(y_{l,t}-C_{l}{\hat{x}}_{k,t}\right)+r_{t}\\ P_{k,t+1} & =A{\left(V_{k,t}^{-1}+S_{k}\right)}^{-1}A^{T}+BB^{T}\\ \mathrm{Find}\; & \theta_{k,t}\;s.t.\;\gamma \left(P_{k,t+1},\theta_{k,t}\right)=c_{k,t}\\ V_{k,t+1} & ={\left(P_{k,t+1}^{-1}-\theta_{k,t}I\right)}^{-1} \end{array}$$**Diffusion step.** Compute at every node *k*:
(33)$${\hat{x}}_{k,t+1}=\sum_{l\in N_{k}}w_{lk}\psi_{l,t+1}$$

### Least Favorable Performance

We show how to evaluate the performance of the previously introduced distributed algorithm with non-uniform local tolerance and diffusion step with respect to the least favorable model solution of the centralized problem in Equation (3). More precisely, we show how to compute the mean and the variance of the prediction error for each node *k* in the network. In [29,34], it is shown that the least favorable model can be characterized through a state-space model over a finite interval [0,T] as follows. Let $\xi_{t}={\left[\;x_{t}^{T}\;e_{t}^{T}\;\right]}^{T}$, where $x_{t}$ is the least favorable state process. Then, the least favorable model takes the form

(34) $$\begin{array}{cc} \xi_{t+1} & ={\overset{ˇ}{A}}_{t}\xi_{t}+{\overset{ˇ}{B}}_{t}\varepsilon_{t}+{\overset{ˇ}{r}}_{t}\\ y_{t} & ={\overset{ˇ}{C}}_{t}\xi_{t}+{\overset{ˇ}{D}}_{t}\varepsilon_{t} \end{array}$$

where $\varepsilon_{t}$ is normalized WGN, independent from ${\hat{x}}_{0}$, and ${\overset{ˇ}{r}}_{t}:={\left[\;r_{t}^{T}\;0\;\right]}^{T}$. Moreover,

$$\begin{array}{ccc} {\overset{ˇ}{A}}_{t}:= & \left[\begin{array}{cc} A & \Gamma_{B}\Gamma_{H_{t}}\\ 0 & A-G_{t}C+\left(\Gamma_{B}-G_{t}\Gamma_{D}\right)\Gamma_{H_{t}} \end{array}\right],\;{\overset{ˇ}{B}}_{t}:= & \left[\begin{array}{c} \Gamma_{B}\Gamma_{L_{t}}\\ \left(\Gamma_{B}-G_{t}\Gamma_{D}\right)\Gamma_{L_{t}} \end{array}\right]\\ {\overset{ˇ}{C}}_{t}:= & \left[\begin{array}{cc} C & \Gamma_{D}\Gamma_{H_{t}} \end{array}\right],\;{\overset{ˇ}{D}}_{t}\Gamma_{D}\Gamma_{L_{t}} \end{array}$$

where $\Gamma_{L_{t}}$ is such that $K_{t}=\Gamma_{L_{t}}\Gamma_{L_{t}}^{T}$,

$$\begin{array}{cc} K_{t}:= & {\left(I-{\left(\Gamma_{B}-G_{t}\Gamma_{D}\right)}^{T}\left(\Omega_{t+1}^{-1}+\theta_{t}I\right)\left(\Gamma_{B}-G_{t}\Gamma_{D}\right)\right)}^{-1}\\ \Gamma_{H_{t}}:= & K_{t}{\left(\Gamma_{B}-G_{t}\Gamma_{D}\right)}^{T}\left(\Omega_{t+1}^{-1}+\theta_{t}I\right)\left(A-G_{t}C\right). \end{array}$$

The matrix $\Omega_{t+1}^{-1}$ is computed from the backward recursion

$$\begin{array}{cc} \Omega_{t}^{-1}={\left(A-G_{t}C\right)}^{T}\left(\Omega_{t+1}^{-1}+\theta_{t}I\right)\left(A-G_{t}C\right) & +\Gamma_{H_{t}}^{T}K_{t}^{-1}\Gamma_{H_{t}} \end{array}$$

where the final point is initialized with $\Omega_{T+1}^{-1}=0$.

Let ${\tilde{x}}_{k,t}=x_{t}-{\hat{x}}_{t,k}$ denote the least favorable state prediction error ${\tilde{x}}_{k,t}$ of node *k* at time *t* using Algorithm 2 or Algorithm 1. Define the vector containing all the errors across the network

$${\tilde{\chi }}_{t}:={\left[\begin{array}{ccc} {\tilde{x}}_{1,t}^{T} & \cdots & {\tilde{x}}_{N,t}^{T} \end{array}\right]}^{T}.$$

Using the same reasonings in [39], it is not difficult to prove that ${\tilde{\chi }}_{t}$ obeys the following dynamics

(35) $$\begin{array}{cc} {\tilde{\chi }}_{t+1} & =A_{t}{\tilde{\chi }}_{t}+B_{t}\varepsilon_{t}+C_{t}e_{t} \end{array}$$

where

$$\begin{array}{cc} A_{t} & :=\left(W^{T}⊗I\right)\left(I⊗A\right){\left(V_{t}^{-1}+S\right)}^{-1}V_{t}^{-1}\\ B_{t} & :=-\left(W^{T}⊗I\right)\left(I⊗A\right){\left(V_{t}^{-1}+S\right)}^{-1}\left(J^{T}⊗I\right)C^{T}R^{-1}DL_{t}+1⊗B\;N_{t}\\ C_{t} & :=-\left(W^{T}⊗I\right)\left(I⊗A\right){\left(V_{t}^{-1}+S\right)}^{-1}\left(J^{T}⊗I\right)C^{T}R^{-1}DH_{t}+1⊗BM_{t}\\ C & :=\mathrm{diag}(C_{1},\ldots ,C_{N})\\ V_{t} & :=\mathrm{diag}(V_{1,t},\ldots ,V_{N,t})\\ S & :=\mathrm{diag}(S_{1},\ldots ,S_{N}), \end{array}$$

$M_{t}\in R^{n\times n}$, $H_{t}\in R^{pN\times n}$, $N_{t}\in R^{n\times (pN+n)}$ and $L_{t}\in R^{pN\times (pN+n)}$ are such that $\Gamma_{H_{t}}={\left[\;M_{t}^{T}\;H_{t}^{T}\;\right]}^{T}$ and $\Gamma_{L_{t}}={\left[\;N_{t}^{T}\;L_{t}^{T}\;\right]}^{T}$. Finally, 1 denotes the vector of ones. Then, we combine Equation (35) with the model for $e_{t}$ in Equation (34):

(36) $$\begin{array}{c} \eta_{t+1}=F_{t}\eta_{t}+G_{t}\varepsilon_{t} \end{array}$$

where $\eta_{t}:={\left[\;{\tilde{\chi }}_{t}^{T}\;e_{t}^{T}\;\right]}^{T}$,

(37) $$\begin{array}{cc} F_{t} & :=\left[\begin{array}{cc} A_{t} & C_{t}\\ 0 & \left(A-G_{t}C\right)+\left(\Gamma_{B}-G_{t}\Gamma_{D}\right)\Gamma_{H_{t}} \end{array}\right]\\ G_{t} & :=\left[\begin{array}{c} B_{t}\\ \left(\Gamma_{B}-G_{t}\Gamma_{D}\right)\Gamma_{L_{t}} \end{array}\right]. \end{array}$$

Taking the expectation of Equation (36), we obtain

(38) $$\begin{array}{c} E\left[\eta_{t+1}\right]=F_{t}E\left[\eta_{t}\right]. \end{array}$$

In view of the fact that $x_{0}$ has mean equal to ${\hat{x}}_{0}$ and ${\hat{x}}_{k,0}={\hat{x}}_{0}$ for k=1…N, it is not difficult to see that $\tilde{E}\left[\eta_{0}\right]=0$. This implies that $\eta_{t}$ is a zero mean stochastic process or, equivalently, all the predictors are unbiased. Next, we show how to derive the variance of the prediction errors. Let $Q_{t}=E\left[\eta_{t}\eta_{t}^{T}\right]$. In view of the fact that $\varepsilon_{t}$ is normalized WGN, by Equation (36), we have that $Q_{t}$ is given by solving the following Lyapunov equation

(39) $$\begin{array}{c} Q_{t+1}=F_{t}Q_{t}F_{t}^{T}+G_{t}G_{t}^{T}. \end{array}$$

We partition $Q_{t}$ as follows:

(40) $$\begin{array}{c} Q_{t}=\left[\begin{array}{cc} P_{t} & H_{t}\\ H_{t}^{T} & R_{t} \end{array}\right] \end{array}$$

where $P_{t}\in R^{Nn\times Nn}$, $H_{t}\in R^{Nn\times n}$ and $R_{t}\in R^{n\times n}$. Notice that $P_{t}$ contains in the main block diagonal the covariance matrices of the estimation error at each node. Accordingly, the least favorable mean square deviation is given by

$${\bar{\mathrm{MSD}}}_{t}:=\frac{1}{N}\sum_{k=1}^{N}{\mathrm{MSD}}_{k,t}=\frac{1}{N}\mathrm{tr}\left(P_{t}\right)$$

where ${\mathrm{MSD}}_{k,t}$ is the variance of the prediction error at node *k*. Finally, we have the following convergence result for the proposed distributed algorithm.

**Proposition** **1.** *Let (A,B) be a reachable pair and $\left(A,C_{k}^{loc}\right)$ be an observable pair for any k. Let W be an arbitrary diffusion matrix satisfying Equation (11). Then, there exists c>0 sufficiently small such that, for any arbitrary initial condition $V_{0}>0$ and $V_{k,0}>0$, the sequence $Q_{t}$, t≥0, generated by Equation (39) converges to $\bar{Q}>0$ over [αT,βT] as T→∞. Moreover, we have $F_{t}\to \bar{F}$, $G_{t}\to \bar{G}$, and $c_{k,t}\to {\bar{c}}_{k}$. In particular, $\bar{Q}$ corresponds to the unique solution of the algebraic Lyapunov equation*

(41) $$\begin{array}{c} \bar{Q}=\bar{F}\bar{Q}{\bar{F}}^{T}+\bar{G}{\bar{G}}^{T} \end{array}$$

*with $\bar{F}$ Schur stable. Accordingly, ${\bar{\mathrm{MSD}}}_{t}$ converges over [αT,βT] as T→∞.*

**Proof.**  First, notice that the observability condition on the pairs $\left(A,C_{k}^{loc}\right)$ implies the observability of (A,C). Since the global model is reachable and observable, the robust centralized Kalman filter converges provided that *c* is sufficiently small (see [43,44]). As a consequence, $V_{t}\to \bar{V}>0$ as t→∞. Accordingly, in view of Equation (17), $K_{z_{t}}\to K_{z}$ and, thus, in view of Equation (22), ${\tilde{K}}_{z_{t}}\to {\tilde{K}}_{z}$. Since $K_{z_{k,t}}$ and ${\tilde{K}}_{z_{k,t}}$ are submatrices of $K_{z_{t}}$ and ${\tilde{K}}_{z_{t}}$, respectively, we have that $K_{z_{k,t}}\to K_{z_{k}}$ and ${\tilde{K}}_{z_{k,t}}\to {\tilde{K}}_{z_{k}}$. Accordingly, in view of Equation (30), we have that $c_{k,t}\to {\bar{c}}_{k}$ where

(42) $$\begin{array}{cc} {\bar{c}}_{k}:= & \frac{1}{2}\left[-\log\left|{\tilde{K}}_{z_{k}}\right|+\log\left|K_{z_{k}}\right|+\mathrm{tr}\left({\tilde{K}}_{z_{k}}K_{z_{k}}^{-1}\right)-\left(n+p_{k}\right)\right]. \end{array}$$

In [30], it has been shown that $V_{t}\to P_{t}$ as c→0, and thus ${\tilde{K}}_{z_{t}}\to K_{z_{t}}$. Since $K_{z_{k,t}}$ and ${\tilde{K}}_{z_{k,t}}$ are submatrices of $K_{z_{t}}$ and ${\tilde{K}}_{z_{t}}$, respectively, we have that ${\tilde{K}}_{z_{k,t}}\to K_{z_{k,t}}$. Accordingly, in view of Equation (30), we have that $c_{k,t}\to 0$ as c→0.

In view of ([43], Proposition 5.3), we conclude that the robust local Kalman filter at node *k* converges because: the local state-space model is reachable and observable; ${\bar{c}}_{k}$ is sufficiently small provided that *c* is sufficiently small as well.

Finally, the remaining part of the proof follows the one in ([39], Section IV-A) (see also [45]). □

It is worth noting that Proposition 1 guarantees that $\bar{Q}$ is bounded because $\bar{F}$ is Schur stable. This means that the prediction errors over the network have finite variance, i.e., the Kalman gains of the local filters are stabilizing. The proof above also shows that, in the case c=0, i.e., the nominal model coincides with the actual one, Algorithm 2 boils down to the distributed Kalman filter with diffusion step proposed in [14].

## 5. Numerical Examples

**Figure 1:**
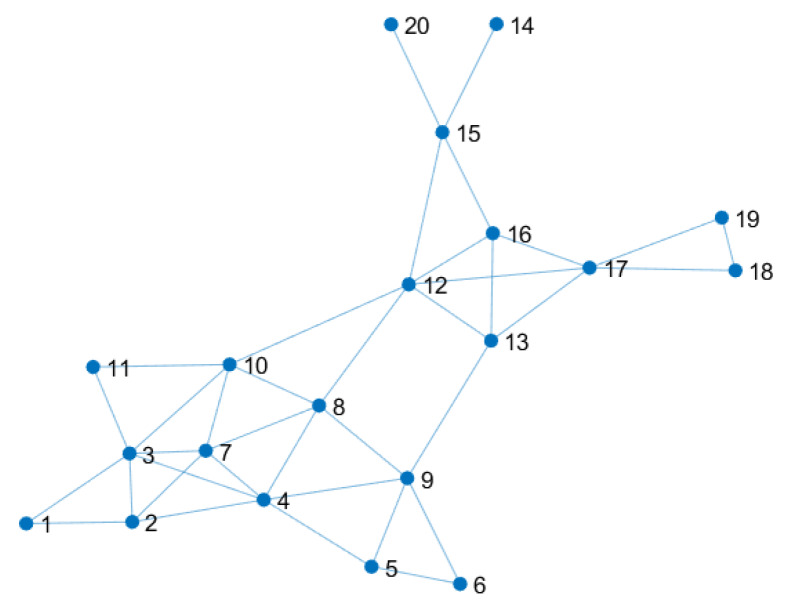
Network of 20 sensors for measuring the noisy positions of the projectile.

In this section, we test the performance of the distributed Kalman filters with uniform versus non-uniform local tolerance. More precisely, we consider the problem in [39] to track the position of a projectile from position observations corrupted by noise and coming from a network of N=20 sensors. The latter is shown in Figure 1.

The model for the projectile motion is

(43) $${\dot{x}}_{t}^{c}=\Phi x_{t}^{c}+r_{t}^{c}$$

where

$$\Phi =\left[\begin{array}{cc} 0 & 0\\ I_{3} & 0 \end{array}\right],$$

$r_{t}^{c}={\left[\begin{array}{cccccc} 0 & 0 & -g & 0 & 0 & 0 \end{array}\right]}^{T}$, with g=10, and $x_{t}^{c}={\left[\begin{array}{cccccc} v_{x,t} & v_{y,t} & v_{z,t} & p_{x,t} & p_{y,t} & p_{z,t} \end{array}\right]}^{T}$, with *v* denoting the velocity and *p* the position in the three spatial components. We discretize Equation (43) by using a sampling time equal to 0.1s. In this way, the model becomes

$$x_{t+1}=Ax_{t}+r_{t}$$

where $x_{t}$ is the sampled version of $x_{t}^{c}$, $A=I_{6}+0.1\Phi$ and $r_{t}=\left(0.1I_{6}+0.1^{2}\Phi /2\right)u_{t}^{c}$. Assuming that every sensor can measure only along two spatial components, the output matrix of the *k*th node can be of the type

$$\begin{array}{c} C_{k}=\left[\begin{array}{cccc} 0 & 0 & 0 & \mathrm{diag}(1,1,0) \end{array}\right],\;C_{k}=\left[\begin{array}{cccc} 0 & 0 & 0 & \mathrm{diag}(1,0,1) \end{array}\right],\;C_{k}=\left[\begin{array}{cccc} 0 & 0 & 0 & \mathrm{diag}(0,1,1) \end{array}\right]. \end{array}$$

Every output matrix is assigned randomly to each node, with the constraint that the local state-space model in Equation (10) associated to each node must be observable for every node to guarantee the convergence of the robust Kalman filters at every node. Thus, if any node violates the constraint, all the output matrices are discarded and reassigned. Then, we choose $B=\sqrt{0.001}I$, $R_{k}=D_{k}D_{k}^{T}=\sqrt{k}PR_{0}P^{T}$, where $R_{0}=0.5\cdot \mathrm{diag}\left(1,4,7\right)$ and *P* is a permutation matrix which is randomly generated for any node. The initial state $x_{0}$ is Gaussian distributed and whose covariance matrix is $P_{0}=I$.

In the numerical simulations, the following Kalman filters are considered: the centralized Kalman filter (KFC); the centralized robust Kalman filter (RKFC); the distributed Kalman filter with diffusion step (KFD) proposed in [14]; the distributed robust Kalman with diffusion step and uniform local tolerance (RKFDU) proposed in [39] and reviewed in Section 3; and the distributed robust Kalman filter with diffusion step and non-uniform local tolerance (RKFDNU) proposed in Section 4. For the distributed filters, the diffusion matrix *W* is defined as

$$w_{lk}=\left\{\begin{array}{cc} \alpha_{k}n_{l}, & \;\mathrm{if}\;l\in N_{k}\\ 0, & \;\mathrm{otherwise} \end{array}\right.$$

where $n_{l}$ represents the total number of neighbors of node *l* while $\alpha_{k}$ is chosen such that Equation (11) holds.

### 5.1. First Example

**Figure 2:**
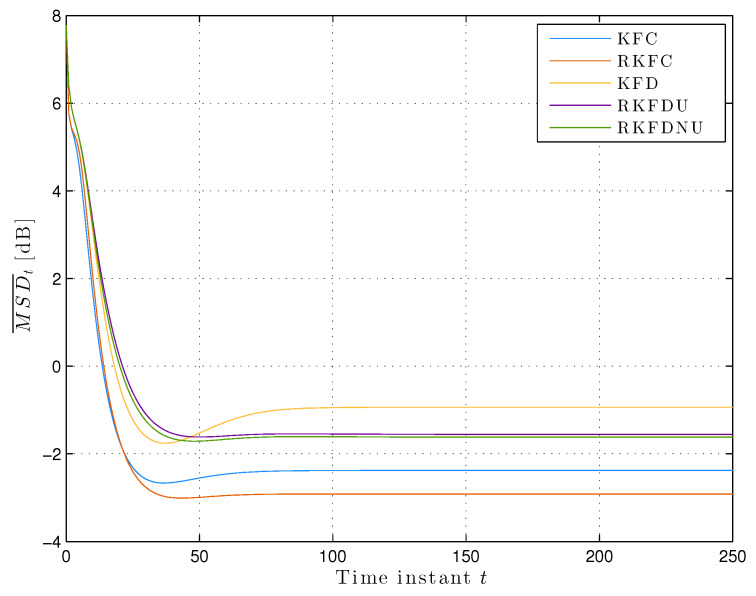
Least favorable mean square deviation across the network with tolerance c=0.02.

We assume that the actual model is contained in the ambiguity set Equation (2) with c=0.02. Figure 2 shows the least favorable mean squared deviation across the network. We notice that ${\bar{\mathrm{MSD}}}_{t}$ converges at the steady-state for all the distributed versions of the Kalman filter. RKFDNU performs slightly better that RKFDU and both perform consistently better than KFD. Finally, all of them perform worse than the centralized versions, and RKFC results the best.

**Figure 3:**
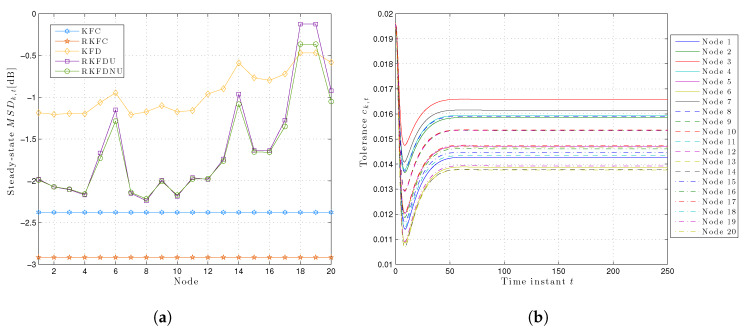
(**a**) Least favorable mean square deviation for each node in the steady-state with tolerance c=0.02. (**b**) Time-variant local tolerances for each node over time with c=0.02.

However, the situation is more salient if we consider the steady-state least favorable ${\mathrm{MSD}}_{k,t}$ for each node (see Figure 3a): RKFDNU performs slightly better than RKFDU for the majority of the nodes. However, there is a clear difference for nodes 18 and 19 which are more susceptible to model uncertainty: RKFDNU performs better than RKFDU.

Figure 3b shows the behavior of the local tolerances $c_{k,t}$ over the time for RKFDNU. As expected, every $c_{k,t}$ converges to a constant value. However, the latter is different from the tolerance *c* of the centralized minimax problem.

**Figure 4:**
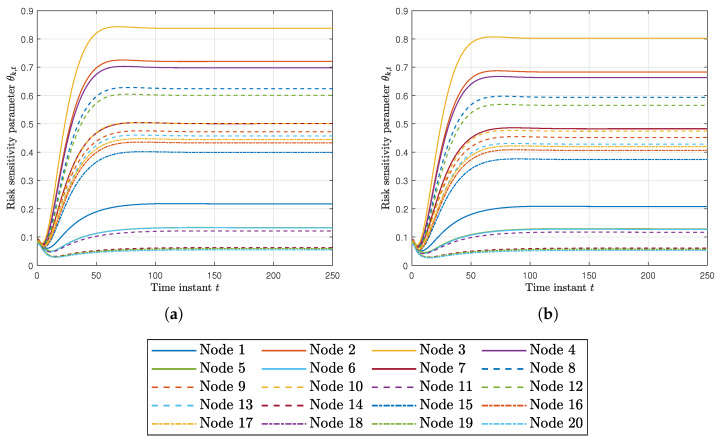
(**a**) Risk sensitivity parameter for each node using RKFDU with tolerance c=0.02. (**b**) Risk sensitivity parameter for each node using RKFDNU with tolerance c=0.02.

Finally, Figure 4a,b shows the risk sensitivity parameters $\theta_{k,t}$ at every node for RKFDU and RKFDNU. We can observe that the risk sensitivity parameters of RKFDU takes larger values than the ones of RKFDNU. Accordingly, the inferior performance of RKFDU is due by the fact that the robust local filters are too conservative.

### 5.2. Second Example

In the second experiment, we consider a larger deviation between the actual model and the nominal one, i.e., we choose c=0.06.

**Figure 5:**
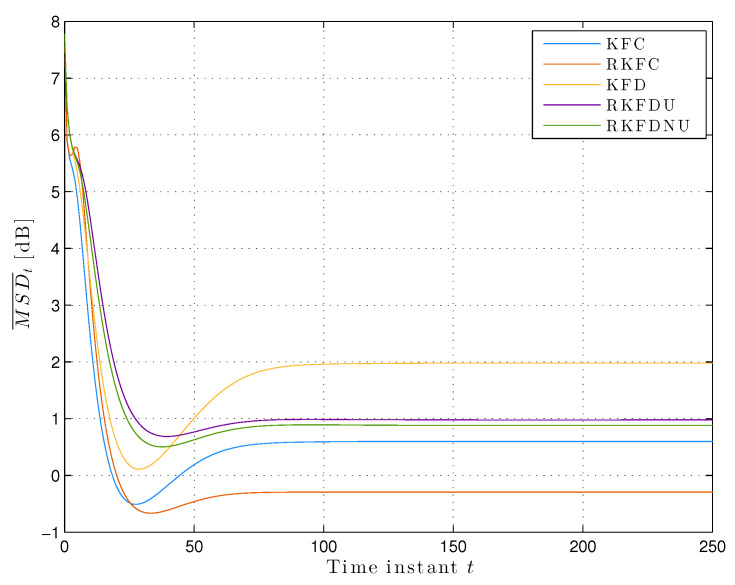
Least favorable mean square deviation across the network with tolerance c=0.06.

**Figure 6:**
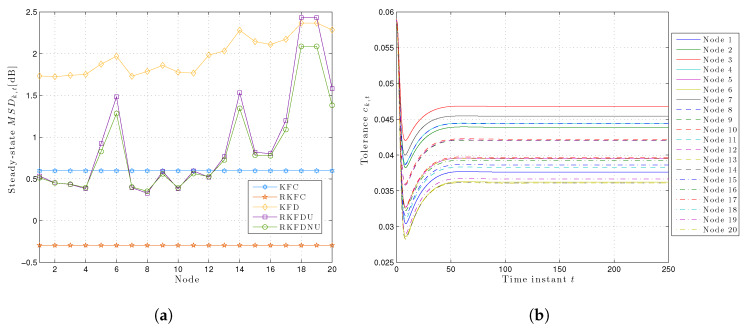
(**a**) Least favorable mean square deviation for each node in the steady-state with tolerance c=0.06. (**b**) Time-variant local tolerances for each node over time with c=0.06.

Figure 5 and Figure 6a show the least favorable mean square deviation across the network and for each node in the steady-state. The situation is similar to the previous one, but the difference among RKFD, RKFDU, and RKFDNU is more evident. In particular, the steady-state value of ${\bar{\mathrm{MSD}}}_{k,t}$ for k=18,19 using RKFDNU is clearly better than the ones corresponding to KFD and RKFDU.

In addition, Figure 6b shows the tolerances $c_{k,t}$ at every node over the time. As expected, the latter are higher than the ones with c=0.02. Indeed, the uncertainty now is greater than before and thus the robust local filters now must be more conservative than before.

**Figure 7:**
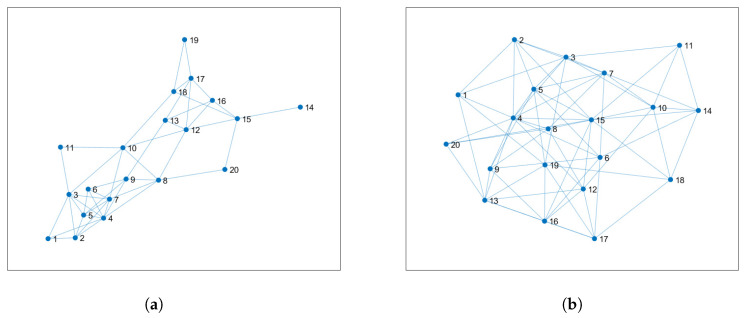
(**a**) Sensor network with density $d_{2}=0.48$. (**b**) Sensor network with density $d_{3}=0.72$.

Finally, we study how the least favorable MSD for each node correlates with the topology of the sensor network. Figure 7a,b shows two additional sensor networks obtained from the original network of Figure 1 by adding connections only to some nodes. More precisely, the density of the original network, i.e., number of connections over all possible connections, is $d_{1}=0.39$; the density of the networks in Figure 7a,b is $d_{2}=0.48$ and $d_{3}=0.72$, respectively.

**Figure 8:**
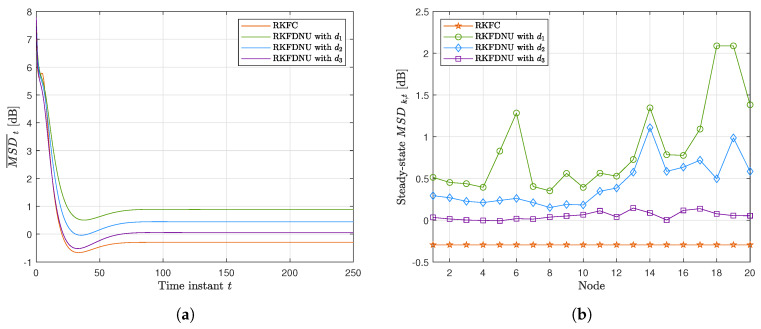
Performance of RKFDNU across the three networks compared with RKFC. (**a**) Least favorable mean square deviation with tolerance c=0.06. (**b**) Least favorable mean square deviation for each node in the steady-state with tolerance c=0.06.

Figure 8a,b shows the results obtained by RKFDNU with the three different sensor networks. As expected, the increase of the degrees of the nodes, and consequently of the connections in the network, reduces the least favorable MSD related to those nodes at steady-state and the total least favorable MSD across the network. In conclusion, by adding edges the performance of RKFDNU tends to the one obtained in the centralized case (RKFC), where the nodes are considered all connected to each other.

## 6. Efficient Algorithm

Proposition 1 suggests a simplified version of Algorithm 2. Indeed, if *c* is sufficiently small, then $c_{k,t}$ converges to ${\bar{c}}_{k}$ in the steady state for every node of the network. Accordingly, the central unit can compute ${\bar{c}}_{k}$ and transmit it to any node once. In this way, the transmission costs are reduced. The resulting procedure is outlined in Algorithm 3.
**Algorithm 3  **Efficient distributed robust Kalman filter with non-uniform local tolerance at time *t*.**Input:**${\hat{x}}_{k,t},V_{k,t},y_{k,t},W={\left[w_{lk}\right]}_{lk}$ with k=1…N**Output:**${\hat{x}}_{k,t+1},V_{k,t+1}$ with k=1…N**Incremental step.** Compute at every node *k*:
(44)$$\begin{array}{cc} \psi_{k,t+1} & =A{\hat{x}}_{k,t}+A{\left(V_{k,t}^{-1}+S_{k}\right)}^{-1}\sum_{l\in N_{k}}C_{l}^{T}R_{l}^{-1}\left(y_{l,t}-C_{l}{\hat{x}}_{k,t}\right)+r_{t}\\ P_{k,t+1} & =A{\left(V_{k,t}^{-1}+S_{k}\right)}^{-1}A^{T}+BB^{T}\\ \mathrm{Find}\; & \theta_{k,t}\;s.t.\;\gamma \left(P_{k,t+1},\theta_{k,t}\right)={\bar{c}}_{k}\\ V_{k,t+1} & ={\left(P_{k,t+1}^{-1}-\theta_{k,t}I\right)}^{-1} \end{array}$$**Diffusion step.** Compute at every node *k*:
(45)$${\hat{x}}_{k,t+1}=\sum_{l\in N_{k}}w_{lk}\psi_{l,t+1}$$

**Figure 9:**
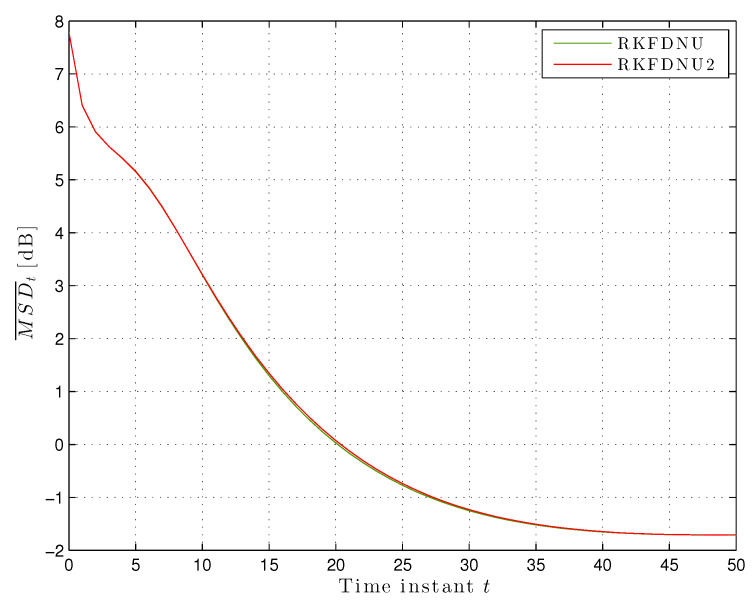
Least favorable mean square deviation across the network with tolerance c=0.02.

**Figure 10:**
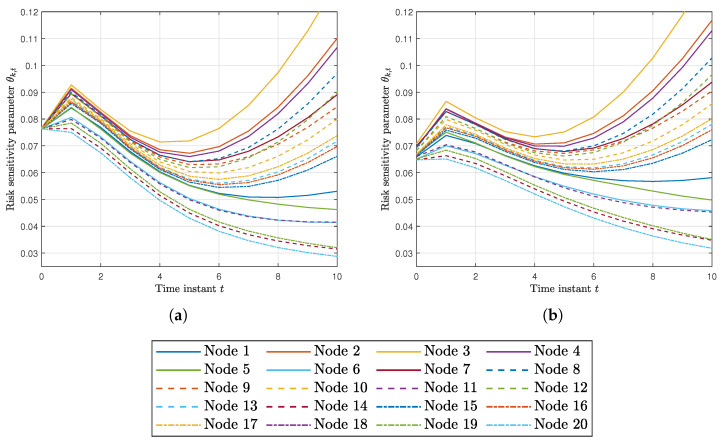
(**a**) Risk sensitivity parameter for each node using RKFDNU with tolerance c=0.02. (**b**) Risk sensitivity parameter for each node using RKFDNU2 with tolerance c=0.02.

We compared this algorithm, hereafter called RKFDNU2, with RKFDNU: the performance in practice is the same. Figure 9 shows their least favorable mean square deviation across the network in the scenario of Section 5.2 in the first 50 time steps. Finally, Figure 10a,b shows the risk sensitivity parameters for RKDFNU and RKFDNU2, respectively: there is a slight difference. However, we saw that such a difference disappears after 20 time steps. We conclude that the efficient scheme RKFDNU2 represents a good approximation of RKFDNU.

Finally, Table 1 summarizes the performance of RKFC, RKFDU, RKFDNU, and RKFDNU2 obtained with tolerance c=0.02. The considered values are the least favorable MSD across the network at steady-state, the average among every node of the tolerances at steady-state, the average among every node of the risk sensitivity parameter at steady-state, and the occurred communications between the central unit and the local nodes in the whole time span. In particular, concerning the communication:

- in RKFDU, the central unit transmits the uniform tolerance to each node at once (at the beginning);
- in RKFDNU, the central unit transmits the local tolerances to each node at every time step;
- in RKFDNU2, the central unit transmits the steady-state local tolerances to each node at once (at the beginning).

## 7. Conclusions

In this article, the problem of distributed robust Kalman filtering for a sensor network is considered. More precisely, we consider a distributed scheme with diffusion step and the intermediate estimate is designed in order to be optimal according to the least favorable model belonging to a prescribed local ambiguity set. The latter is a ball about the local nominal model and the radius of this ball is the local tolerance. In this paper, we propose an algorithm in which the local tolerance of each node is different and suitably computed by the central unit. We also consider a more efficient implementation of the algorithm where the central unit computes and transmits the steady-state local tolerances for every node at once. In this way, the communication between the central unit and the local nodes is reduced. Through some numerical examples, we showed that the proposed algorithm performs better than the one with a uniform local tolerance across the network. 

## Figures and Tables

**Table 1:** Summary of the performance of the different algorithms with tolerance c=0.02.

	RKFC	RKFDU	RKFDNU	RKFDNU2
**MSD [dB]**	−2.9174	−1.5553	−1.6182	−1.6182
**Average tolerance**	0.02	0.02	0.0144	0.0144
**Average risk sensitivity parameter**	1.3366	0.3764	0.3576	0.3576
**Communication requirement**	N.A.	One at the beginning	Every time step	One at the beginning
